# The Fires of Isengard Have Spread: *Serratia sarumanii* Is the Dominant Species in Clinical Isolates of the “*Serratia marcescens* Complex”

**DOI:** 10.3390/pathogens15020140

**Published:** 2026-01-28

**Authors:** Levin Joe Klages, Julia Hassa, Tobias Busche, Olaf Kaup, Christiane Scherer, Claudia Christine Freytag, Thorsten Kaiser, Jörn Kalinowski, Christian Rückert-Reed

**Affiliations:** 1 Microbial Genomics and Biotechnology, Center for Biotechnology (CeBiTec), Bielefeld University, 33615 Bielefeld, Germany; lklages@uni-bielefeld.de (L.J.K.); joern@cebitec.uni-bielefeld.de (J.K.); 2 Center for Biotechnology (CeBiTec), Bielefeld University, 33615 Bielefeld, Germany; julia.hassa@uni-bielefeld.de (J.H.); tobias.busche@uni-bielefeld.de (T.B.); 3 Medical School OWL, Bielefeld University, 33615 Bielefeld, Germany; olaf.kaup@klinikumbielefeld.de (O.K.); c.scherer@ladr.de (C.S.); claudiachristine.freytag@klinikum-lippe.de (C.C.F.); thorsten.kaiser@uni-bielefeld.de (T.K.); 4 Institute of Laboratory Medicine, Microbiology and Transfusion Medicine, University Medical Center OWL, Bielefeld Hospital, 33615 Bielefeld, Germany; 5 Institute of Laboratory Medicine, Microbiology and Hygiene, University Medical Center OWL, Evangelisches Klinikum Bethel, 33615 Bielefeld, Germany; 6 Institute of Laboratory Medicine, Microbiology and Clinical Pathobiochemistry, University Medical Center OWL, Klinikum Lippe, 32756 Detmold, Germany

**Keywords:** *Serratia*, genomics, clinical relevance, pathogen

## Abstract

Recently, a new species, *Serratia sarumanii*, was described, belonging to a group of strains previously identified as *Serratia marcescens* in routine clinical analyses. It was shown that the identification of *S. marcescens* isolates by biochemical testing, mass spectrometry, or 16S rRNA gene sequencing was insufficient to resolve the ‘*S. marcescens* complex’, while sampling point analysis revealed that many genomes assigned to the *S. sarumanii* cluster were associated with a clinical context. Thus, here the clinical relevance and local as well as global distribution of *S. sarumanii* is analyzed. In total, 21 strains from three hospitals in Eastern Westphalia-Lippe (OWL), previously identified as *S. marcescens* and potential causative agents from severe bacterial infections, were analyzed by genome sequencing and species identification. It could be shown that only one isolate was confirmed as *S. marcescens*, whereas 10 of the 21 isolates were identified as *S. sarumanii*, indicating that *S. sarumanii* is the dominant representative of the “*Serratia marcescens*” complex in hospitals in OWL. To analyze the global species distribution, all *Serratia* genomes available in GenBank were reclassified. About one-third of these genomes were identified as *S. sarumanii*, indicating *S. sarumanii* as the most dominant *Serratia* species in clinical settings around the world.

## 1. Introduction

The genus *Serratia*, belonging to the order *Enterobacterales*, to date includes 24 species of Gram-negative, facultative anaerobic, rod-shaped bacteria [1,2,3]. The type strain of the genus is *Serratia marcescens*, which can cause infections in humans, animals, and insects [4]. *S. marcescens* is also well known for its red colony phenotype, caused by the secondary metabolite and pigment prodigiosin [1,5]. Interestingly, *S. marcescens* was used from the 1940s to the mid-1960s by the U.S. government as a tracer in medical experiments and biological warfare test agents before its pathogenicity was known [4]. *Serratia* spp. cause a wide range of asymptomatic and symptomatic infections in humans, such as respiratory, urinary, surgical wound, and bloodstream infections, as well as meningitis and keratitis [3,6].

The clinical identification of such pathogens usually relies on phenotypical identification, biochemical assays, or mass spectrometry (MS) [7,8,9]. Therefore, mass spectrometry platforms like MALDI Biotyper (Bruker Daltonics) and biochemical analysis platforms like VITEK-MS (bioMérieux) are registered for clinical uses [7]. However, the clinical identification via VITEK2 and even MALDI-TOF-MS is not always sufficient for the correct species identification of certain clinically relevant pathogens, e.g., *Serratia sarumanii* [10]. As a result, *S. marcescens* was grouped in the ‘*S. marcescens* complex’ (SMC) together with closely related *Serratia* species, such as *S. nematophilia* and *S. ureilytica* [11] as well as *S. bockelmannii* and *S. nevei* [12]. The SMC can, currently, only be reliably subdivided with whole genome-based methods.

In our previous publication, we described the discovery of the new species *Serratia sarumanii* [10]. The species was previously classified as ‘*S. marcescens’* and is therefore also part of the SMC. Furthermore, it has been shown that most strains in the Genome Taxonomy Database (GTDB) that were assigned to the new species originate from a clinical setting, raising the questions of *Serratia sarumanii*’s role in the SMC, both regionally and globally. To address these questions, *Serratia* isolates collected in three hospitals in Eastern Westphalia-Lippe (OWL) were analyzed using overall genome-relatedness indices as well as marker gene-based phylogenies. The approach was then expanded to reclassify all genomes submitted as *Serratia* species in the NCBI nucleotide database.

## 2. Materials and Methods

### 2.1. Clinical Sample Collection, DNA Isolation, and Initial Species Identification

A total of 21 samples were collected from patients in the context of diagnosed sepsis cases at three University Medical Center OWL hospitals: Klinikum Bielefeld (KBM), Evangelisches Klinikum Bethel (EvKB), and Klinikum Lippe (KL). The sampling was performed as standard clinical routine directly from blood, wound swabs, peritoneal wash fluent, or urine culture.

The cultivation and sample preparation of the eight samples originating from the hospital KBM were performed as described in a recent publication by Klages et al. [10].

All other samples were cultivated in a liquid blood culture medium at 37 °C. After the cultivation in blood culture, the samples were further grown on different solid media, and single colonies were selected for further analysis. Species identification of the cultivated microorganisms was performed in the hospital KBM biochemically on the VITEK2 system (bioMérieux, Marcy-l’Étoile, France) with the Gram-negative identification card (GN) and at the two other hospitals additionally via mass spectrometry on the MALDI Biotyper (Bruker Daltonics, Bremen, Germany).

For MALDI-TOF analysis, the α-cyano-4-hydroxycinnamic acid matrix was dissolved in 50% acetonitrile and 2.5% trifluoroacetic acid (TFA). For the measurement, 1 μL each of the sample extract, formic acid, and the prepared matrix solution was consecutively pipetted onto a steel target plate (Bruker Daltonics, Bremen, Germany) and allowed to dry before applying the next solution. The target plate was then placed into the MALDI Microflex LT. The results were processed with the MBT compass IVD (Bruker Daltonics, Bremen, Germany).

Subsequently, at *KBM*, DNA extraction was performed using the GenoXtract device (HAIN Lifescience GmbH (now Bruker Daltonics, Bremen, Germany)), applying the ‘GXT NA Extraction as described in the recent publication by Klages et al. [10].

Genomic DNA isolation at *KL* and *EvKB* was performed using the Maxwell RSC Cultured Cells DNA Kit on the Maxwell RSC device (Promega, Madison, WI, USA), according to the manufacturer’s protocol.

### 2.2. Whole Genome Sequencing and Genome Assembly

Nanopore sequencing (Oxford Nanopore Technologies, ONT, Oxford, UK) was performed as described by Klages et al. [10]. Briefly, the DNA obtained from the cultivated organisms was used for sequencing library preparation, applying the ONT SQK-LSK112 kit with the native barcodes SQK-NBD112.24, according to the manufacturer’s protocol. The libraries were sequenced on an R10.4.1 flow cell using the GridION sequencing platform, and the resulting sequences were subsequently basecalled with either GUPPY v6.2.11, v6.2.8, or v6.3.7 in super high accuracy (SUP) mode. To obtain the whole genome sequences, the raw sequencing data were assembled using FLYE v2.9-b1768 [13] and manually curated using Bandage v. 0.8.1 [14] to obtain complete genome assemblies. The genomes were annotated using PGAP 2025-05-06.build7983 [15] and are available via BioProject PRJNA1274668 (National Center for Biotechnology Information, NCBI, Bethesda, MD, USA).

### 2.3. Overall Genome-Relatedness Indices

The genome sequences were uploaded to the TYpe strain Genome Server (TYGS, https://tygs.dsmz.de, 14 August 2023) for a whole genome dDDH-based taxonomic analysis [16,17]. Information on nomenclature, synonymy, and the associated taxonomic literature was provided by TYGS’s sister database, the List of Prokaryotic names with Standing in Nomenclature (LPSN, https://lpsn.dsmz.de, 14 August 2023) [17,18].

For the phylogenetic analysis, all genomes of *Serratia* species were downloaded from the National Center for Biotechnology Information (NCBI) repository [accessed at 29 January 2025] and used for a comprehensive genome-wide comparison with GTDB-tk v.2.4.0 [19] and GTDB [20] release 226, applying the workflows classify_wf and de_novo_wf with the parameters–taxa_filter g__Serratia–outgroup_taxon “s__Yersinia pestis”.

## 3. Results

### 3.1. Prevalence of Species Belonging to the Serratia marcescens Complex (SMC) Within the Regional Clinical Isolates

To determine the prevalent *Serratia* species of the SMC in the regional hospitals, samples were taken from patients in the three cooperating facilities (Klinikum Bielefeld, Ev. Klinikum Bethel, and Klinikum Lippe) over the course of one year (mid-2021 to mid-2022). From these samples, bacterial isolates were cultivated on blood agar as described above.

In total, 21 bacterial isolates from sepsis patients were identified as “*Serratia marcescens*” (Table 1) using routine clinical identification methods. The majority of these 21 isolates originated from blood samples, but some were also derived from wound swabs, urine, or peritoneal lavage in the three hospitals (Table 1).

In general, the subdivision of SMC strains identified via biochemical assays or mass spectrometry can partially be performed based on the phenotype of this pathogen during cultivation, as cultures of *S. marcescens* and *S. nematodiphila* show a blood-red color, while the other members are white. As only one of the 21 “*S. marcescens*” cultures showed a red colony phenotype, this was a first indication that the clinically relevant regional strains belong to the latter group.

### 3.2. Genome-Based Classification of the Regional SMC Strains

To resolve the taxonomy of these SMC strains, their genomes were sequenced, assembled, and used for taxonomic classification. As we recently demonstrated [10], 16S-based comparisons are insufficient to resolve species in the SMC. This is validated here, as 16S rRNA comparisons for the 21 isolates based on the Type Strain Genome Server (TYGS) were also inconclusive (Supplementary Figure SA).

**Figure 1:**
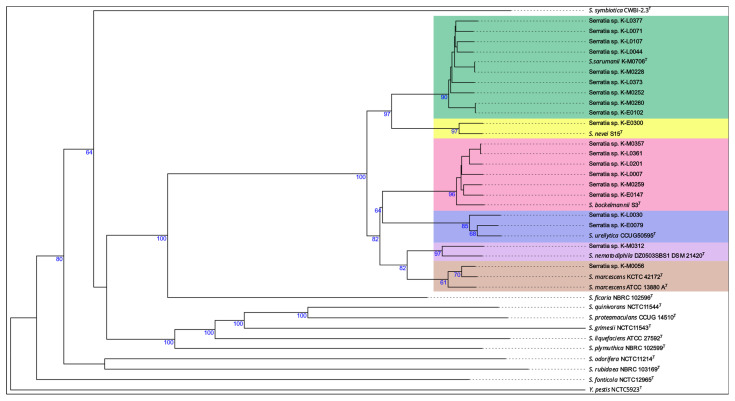
Distance tree of the dDDH values of the genomes of the 21 clinical cultures. The genomes of the 21 clinical cultures were compared to the genomes of all *Serratia* type strains and *Yersinia pestis* as an outgroup. The different species clusters and subspecies clusters are color-coded, with one color for each species and subspecies. The tree was inferred with FastME 2.1.6.1 from GBDP distances calculated from genome sequences. The branch lengths are scaled in terms of the GBDP distance formula d_5_. The numbers above the branches are GBDP pseudo-bootstrap support values > 60% from 100 replications, with an average branch support of 66.4%. The tree was rooted at the midpoint.

The first overall genome-relatedness index used to identify the species was to calculate the digital DNA-DNA hybridization (dDDH) values (Figure 1). These correlate to the classic DNA-DNA hybridization method and were also performed via the TYGS in an all-versus-all manner (Supplementary Table SA). Due to the fact that the genome of the *S. nevei* type strain was missing in the TYGS, we manually included it (GCA_037948395.1; available online: https://www.ncbi.nlm.nih.gov/datasets/genome/GCA_037948395.1/ (accessed on 30 August 2022)). The analysis identified one genome as belonging to *S. marcescens*. Furthermore, six genomes were identified as *S. bockelmannii*, one as *S. nematodiphila*, two genomes as *S. ureilytica*, one as *S. nevei*, and ten genomes as belonging to the recently published species *S. sarumanii* (Table 2).

Remarkably, the TYGS marks *S. nematodiphila* as a potential subspecies of *S. marcescens*, which aligns with the phenotypic similarity of the two species based on the presence of the prodigiosin gene cluster. Another noteworthy observation is the extremely high degree of similarity between the strains K-M0260 and K-E0102, as well as between strains K-M0706 and K-M0228, each being reported with 100.0% dDDH, indicating they might be identical. A closer analysis of both pairs using blastn revealed that K-M0706 and K-M0228, identified in the same hospital, are indeed basically identical, with just 114 mismatches. In contrast, despite their similarity, K-M0260 and K-E0102 were collected from different hospitals and differ by about 370 single nucleotide polymorphisms (SNPs) and deletion/insertion polymorphisms (DIPs), as well as in the presence of 5 larger regions in each genome (between 6 kb and 66 kb for K-M0260 and between 15 kb and 72 kb for K-E0102) that are absent in the other genome. This is noteworthy because a 100.0% dDDH should indicate basically identical genomes, yet they contain approximately 181 and 202 kbp of unique sequences, respectively. Additionally, in contrast to K-E0102, two plasmids with a size of 15.7 and 76.5 kbp were found in K-M0260.

For visualization, the genomes of the 21 samples, the genomes of all *Serratia* type strains, as well as the *Yersinia pestis* type strain as an outgroup, were used for analysis using the TYGS with distance formula d_5_ [21]. The species belonging to the SMC form a distinct group within the tree that is clearly separated from the other *Serratia* (Figure 1). Within the cluster, the *S. marcescens* and *S. nematodiphila* type strains, as well as K-M0312 and K-M0056, cluster even closer together, indicating that *S. nematodiphila* might be a subspecies of *S. marcescens*, as their dDDH value of 73.6% is above the cutoff of 70%.

To verify the dDDH distance-based tree, the genomes used for the TYGS-based analyses were also used for a taxonomic analysis applying GTDB-tk v2.4.0 and GTDB and release 226. The results of the classify_wf workflow as well as the de_novo_wf workflow closely support the results reported by the TYGS (Supplementary Table SB, Supplementary Figure SB). As indicated by the TYGS, the ANI and alignment fraction (AF) of *nematodiphila* and *S. marcescens* indicate that the former might be considered to be a subspecies of the latter (96.8% ANI, 91.0% AF).

Besides the chromosomes, we were able to identify seven distinct plasmids in the 21 samples (Supplementary Table SC). While five of them were found in a single sample and the fifth in two, one plasmid of 83.6 kbp was observed to be present in a total of ten samples, nine of which carried an almost identical version (while in the tenth, it is about 7 kbp larger due to two additional regions of about 11 kbp total and the loss of a 4 kbp region). Interestingly, those ten samples consist of seven *S. sarumanii*, two *S. bockelmannii*, and one *S. ureilytica*, indicating that this plasmid has the capability to be transferred between the different species. Indeed, based on the annotation, almost half of the plasmid encodes for genes known to be involved in mobilization and plasmid transfer (see annotated GenBank entries, IDs listed in Supplementary Table SC).

### 3.3. Analysis of the Abundance of Serratia sarumanii Within All Publicly Available Serratia Genome Sequences

The frequent occurrence of *S. sarumanii* (10 out of 21 samples) and *S. bockelmannii* (6 out of 21 samples) is intriguing due to their recent description as valid species, resulting in the obvious question of their distribution on a global scale.

To address this question, all *Serratia* genomes in the NCBI genomes database (accessed on 29 January 2025), 4172 in total, were downloaded and, together with the genomes of the 21 samples in this study, classified using GTDB-tk. Based on the classify_wf workflow, most genomes in the database belong to the SMC (3574 = 85.7%), while other *Serratia* and those assigned to other genera are a small minority (433 *Serratia* sp., 115 *Chania* sp, 33 Serratia_B, 13 Serratia_F). Surprisingly, more than a third of all genomes belong to *S. sarumanii* (1453 = 34.8%), which equals 40.7% of the SMC genomes. The following most abundant species, *S. nevei*, contributes just half that number (729 = 17.5% total/20.4% SMC), followed by *S. ureilytica* (460 = 11.0%/12.9% SMC) and *S. bockelmannii* (351 = 8.4%/9.8% SMC), each contributing about half of that, respectively. Genomes of the name-giving *S. marcescens* only make up 261 entries (6.3%/7.3% SMC), barely more than those from *S. nematodiphila* (246 = 5.9%/6.9% SMC).

**Figure 2:**
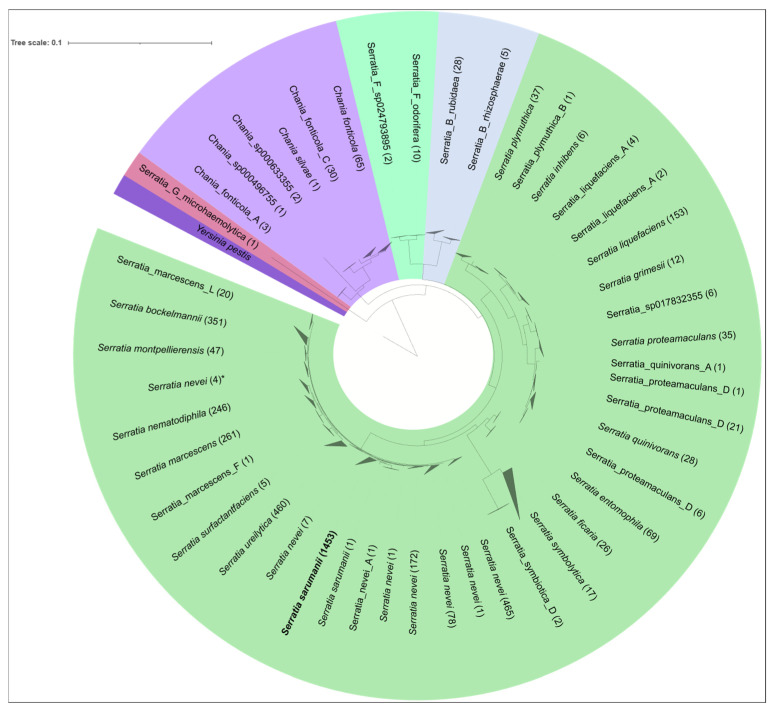
Phylogenetic tree of the genus Serratia with all samples from the NCBI database based on GTDB-TK calculations. Classification of the different branches into the known *Serratia* species was manually performed using GTDB-TK classification and dDDH calculation of different representatives of each branch. The visualization of the tree was performed using iTOL. The coloration is based on the genus classification of GTDB-TK. * indicates the *S. nevei* “clade” not clustering with the one containing the type strain.

To obtain a clearer understanding of the data, a phylogenetic tree was calculated using the de_novo_wf workflow of GTDB-TK, which was then visualized with iTOL (https://itol.embl.de/, 6 May 2025) (Figure 2). Except for a fair number of *S. nevei* genomes (and a single *S. sarumanii* genome) that form multiple clades besides those belonging to the respective type species within the SMC clade, all genomes fall into distinct clades with that of the corresponding type species (Figure 2). The split within *S. nevei* was already observed by Klages et al. [10], who described three distinct groups based on average nucleotide identity (ANI) and alignment fraction (AF) for this species.

Also of interest is the observation that 9 of the 10 *S. sarumanii* are distinct isolates, with only one pair (K-M0706 and K-M0228, both isolated at the same hospital) showing so little difference that they are likely part of an infection chain, either directly from patient to patient or from a common, unidentified source. Given that the two patients were admitted three months apart, the latter explanation is more likely. Even more interesting is a second pair of strains, K-M0260 and K-E0102, which were isolated from two different hospitals eight months apart. They differ by just about 370 SNPs/DIPs, but each harbors five larger regions (6 to 72 kbp in length), containing a total of 181 and 202 kbp of unique sequences, respectively. In addition, K-M0260 harbors two plasmids of 15.7 and 76.5 kbp. This indicates that even if they are derived from a common source, which is likely, they each either gained a significant amount of information (3.5 to 5.5% of their total genome size) via HTG events or lost it while diverging from the common ancestor. Given its prevalence in the databases, another important piece of information regarding the clinical significance of the various species within the SMC is their actual “origin” (i.e., the source material the respective strain was isolated from). Unfortunately, this information is often not available; e.g., in [10], the isolation source was present for 87.0% of the genomes of *S. sarumanii* strains. While this restricts a comprehensive analysis, we focused on several studies addressing the species distribution within the SMC [11,12,22,23,24] that included in-depth information on the source of the respective samples. In all of them, the authors observed a large group of genomes that was clearly separate from the type species validly described at the respective time. We used either the assemblies presented in these studies or, if only raw data were available from SRA, we created draft assemblies with SPAdes v3.13.0 [25] using standard parameters. Running the classify_wf and the de_novo_wf workflow, we could show in all these studies that the “unknown” group consisted of strains belonging to *S. sarumanii* (Supplementary Table SD, Supplementary Figure SC). For example, 166 of 455 isolates (36.5%) in the outbreak investigation by Taxt et al. [24] belong to *S. sarumanii*. Likewise, in the studies of [11,12], the largest cluster, consisting of 47 of 225 samples (20.9%), respectively, 41 of 165 samples (24.8%), is *S. sarumanii*.

We also used BLAST v2.15.0+ to search for the plasmid we identified to be present in several species and were able to detect it (i.e., >75% coverage of the query sequence) in 48 assemblies of *Serratia* spp. in the NCBI database (Supplementary Table SE). Again, it was found in a variety of species, consisting of 23 *S. sarumanii*, 16 *S. bockelmannii*, 4 *S. nevei*, 3 *S. ureilytica*, and 2 *S. nematodiphila*, based on our classification above.

## 4. Discussion

The genome-based analysis of 21 “*Serratia marcescens*” isolates from clinical samples associated with bacterial infections in three local hospitals in *OWL* revealed that they belong predominantly to *Serratia* species other than *S. marcescens.* While this is hardly a novel observation, having been reported, e.g., by Ono et al. [11] and Aracil-Gisbert et al. [12], we are the first to identify the dominant species as *S. sarumanii*. While the number of occurrences in the regional hospitals is low, with just 21 isolates of the SMC in one year, the number of *S. sarumanii* isolates among them is still significant (10 out of 21 isolates = 47.6%). Of the remaining 11, only one really belongs to the species *S. marcescens*, while the rest comprises *S. bockelmannii* (6), *S. ureilytica* (2), *S. nevei* (1), and *S. nematodiphila* (1), although dDDH, ANI, and AF values support that *S. nematodiphila* should be reclassified as a subspecies of *S. marcescens*.

Analysis of all assembled *Serratia* genomes in GenBank (NCBI) confirms the dominance of *S. sarumanii* among the strains of scientific interest, as approximately 40% of the genomes from species currently classified as belonging to the SMC originate from *S. sarumanii*. This “dominance” can be attributed to a high clinical significance, both in terms of prevalence in clinical samples overall and in its dissemination in patient-derived samples [11,12,24]. Besides this prevalence in samples overall, *S. sarumanii* is noteworthy since, in contrast to most other members of the SMC in larger studies, the samples are usually derived from patients [22], predominantly from blood samples [10,11]. This coincides with *S. sarumanii* being often found in intensive care units [12,22], indicating that this species either is especially problematic for patients in already critical condition or is responsible for said critical condition. Another marker that indicates the increased pathogenicity of *S. sarumanii* compared to the second major *Serratia* species in a clinical context, *Serratia nevei*, is the smaller average genome size of the former: 5.1 ± 0.1 Mbp for *S. sarumanii* and 5.7 ± 0.2 Mbp for *S. nevei*. While the actual cause(s) for this correlation are still unclear, it can be observed regardless of the taxonomic level [26].

Surprisingly, though the strains we identified to be *S. sarumanii* harbor fewer resistance markers on average than *S. neveii*, the latter often occurs in hospital environments and has been shown to persist for extended periods in hospital sinks, acquiring highly conserved plasmids that carry multiple resistance-conferring genes [12]. This reservoir function poses a significant risk in hospital hygiene and patient safety, as recent findings by Zhang et al. [27] and Khalifa et al. [23] indicate that the relative absence of resistance markers is counteracted by the apparent ability of “*S. marcescens*”, identified by us as *S. sarumanii*, to quickly acquire and transfer resistance genes, both intra- and interspecies.

In combination with the findings of [11], who identified additional *Serratia* species in hospital settings harboring multiple resistance genes, and those of Aracil-Gisbert et al. [12], who report *S. neveii* and a group of strains we identified as *S. sarumanii* as the most frequently detected *Serratia* species in patient samples, a deeply concerning picture emerges. *S. sarumanii* appears particularly dangerous, not only due to its clinical prevalence but also because it might serve as a center for resistance gene acquisition and dissemination [27]. Furthermore, Khalifa et al. [23] confirmed that *S. nevei* and the group of strains we showed to be S. *sarumanii* are the most common *Serratia* in hospitals harboring several resistance genes, showing that all carbapenem-resistant clinical strains from the *Serratia marcescens* complex can be assigned to the species *S. nevei* and *S. sarumanii*. As a possible route of transfer, plasmids that replicate in different *Serratia* species are a strong possibility. For example, the 83.6 kbp plasmid, which was found in 10 out of 21 samples in our study, was also found in about 1.4% of SMC strains in the NCBI database (48 out of 3574). While this plasmid lacks resistance markers (at least in our samples), those could be easily acquired via horizontal gene transfer (HTG). The occurrence of several HTG events in *S. sarumanii*, possibly in a very short time (as measured by SNP occurrence), is evidenced by the strains K-M0260 and K-E0102, each of which gained about 5% additional genetic information in 5 islands each.

## 5. Conclusions

This study demonstrates that genome-based species identification provides a more accurate picture of clinically relevant species within the ‘*Serratia marcescens* complex’ (SMC) than the conventional clinical classification. Our analysis of clinical isolates from regional hospitals, supported by comparative data from publicly available genomes, reveals that several *Serratia* species contribute to infections previously attributed to *S. marcescens*. In particular, *S. sarumanii* emerges as the predominant species, showing a strong association with patient-derived samples, intensive care units, and a high potential for acquiring and spreading resistance genes. These findings underscore the clinical importance of precise taxonomic resolution for epidemiological surveillance, infection control, and antimicrobial stewardship.

Given the clinical dominance of *S. sarumanii*, capacity for resistance gene transfer, strong association with hospital-acquired infections, and scarcity of *S. marcescens* in clinical isolates, we propose that the term *Serratia marcescens* complex (SMC) is no longer scientifically appropriate or optimal for use in clinical or genomic contexts. Based on the presented evidence, we propose to rename the SMC to *Serratia sarumanii* complex (SSC), to reflect its true composition and allow for more accurate identification, surveillance, and treatment strategies.

## Figures and Tables

**Table 1:** List of the isolated human clinical samples that were identified as Serratia marcescens using clinical identification methods.

Sample Name	Hospital of Origin ^1^	Collected From	Identification Method ^2^	Source
K-E0079	EvKB	blood	MS, BA	this study
K-E0102	EvKB	peritoneal lavage	MS, BA	this study
K-E0147	EvKB	blood	MS, BA	this study
K-E0300	EvKB	blood	MS, BA	this study
K-L0007	KL	blood	MS, BA	this study
K-L0030	KL	blood	MS, BA	this study
K-L0044	KL	blood	MS, BA	this study
K-L0071	KL	blood	MS, BA	this study
K-L0107	KL	blood	MS, BA	this study
K-L0201	KL	blood	MS, BA	this study
K-L0361	KL	blood	MS, BA	this study
K-L0373	KL	punctation (aszites)	MS, BA	this study
K-L0377	KL	blood	MS, BA	this study
K-M0056	KMB	blood	BA	this study
K-M0228	KMB	wound swab	BA	[10]
K-M0252	KMB	urine	BA	[10]
K-M0259	KMB	wound swab	BA	this study
K-M0260	KMB	wound swab	BA	[10]
K-M0312	KMB	blood	BA	this study
K-M0357	KMB	bronchial secret	BA	this study
K-M0706	*KMB*	wound swab	BA	[10]

^1^ *KMB* = *Klinikum Bielefeld*, *EvKB* = *Evangelisches Klinikum Bethel*, *KL* = *Klinikum Lippe*. ^2^ MS = mass spectrometry, BA = biochemical assays.

**Table 2:** Species assignment of the genomes of 21 strains belonging to the “ *Serratia marcescens* complex” (SMC).

Sample Name	Assigned Species	dDDH [%] to Closest Type Strain ^1^	ANI [%] ^2^	AF [%] ^3^
K-E0079	*Serratia ureilytica*	92.0	99.02	93.70
K-E0102	*Serratia sarumanii*	90.4	98.80	93.00
K-E0147	*Serratia bockelmannii*	90.3	98.75	91.90
K-E0300	*Serratia nevei*	91.5	98.86	90.10
K-L0007	*Serratia bockelmannii*	90.1	98.61	90.40
K-L0030	*Serratia ureilytica*	88.5	98.53	93.60
K-L0044	*Serratia sarumanii*	93.4	99.03	96.80
K-L0071	*Serratia sarumanii*	93.2	99.08	96.80
K-L0107	*Serratia sarumanii*	93.1	99.02	96.30
K-L0201	*Serratia bockelmannii*	90.5	98.73	90.60
K-L0361	*Serratia bockelmannii*	90.9	98.80	91.40
K-L0373	*Serratia sarumanii*	92.0	98.92	95.00
K-L0377	*Serratia sarumanii*	92.0	98.92	95.20
K-M0056	*Serratia marcescens*	94.9	98.75	94.60
K-M0228	*Serratia sarumanii*	100.0	100.00	99.90
K-M0252	*Serratia sarumanii*	91.4	98.80	95.80
K-M0259	*Serratia bockelmannii*	90.5	98.75	92.00
K-M0260	*Serratia sarumanii*	90.0	98.58	93.30
K-M0312	*Serratia nematodiphila*	84.9	98.24	88.00
K-M0357	*Serratia bockelmannii*	90.7	98.75	91.20
K-M0706	*Serratia sarumanii*	100.0	100.00	100.00

^1^ TYGS-based dDDH values using formula d_4_ [16]. ^2^ GTDB-TK-based average nucleotide identity (ANI) values [19]. ^3^ GTDB-TK-based alignment fraction (AF) values [19].

## Data Availability

All sequencing, assembly, and annotation data produced in this study are available via BioProject PRJNA1274668 Available online: https://www.ncbi.nlm.nih.gov/bioproject/PRJNA1274668 (accessed on 18 December 2025). Specifically, the genomes for the 21 strains are available via the GenBank IDs CP196503–CP196504 Available online: https://www.ncbi.nlm.nih.gov/nuccore/?term=CP196503:CP196504%5baccn%5d (accessed on 18 December 2025). (K-E0079), CP124754 Available online: https://www.ncbi.nlm.nih.gov/nuccore/CP124754 (accessed on 18 December 2025). (K-E0102), CP196505–CP196506 Available online: https://www.ncbi.nlm.nih.gov/nuccore/?term=CP196505:CP196506%5baccn%5d (accessed on 18 December 2025). (K-E0147), CP196510–CP196511 Available online: https://www.ncbi.nlm.nih.gov/nuccore/?term=CP196510:CP196511%5baccn%5d (accessed on 18 December 2025). (K-E0300), CP196512–CP196513 Available online: https://www.ncbi.nlm.nih.gov/nuccore/?term=CP196512:CP196513%5baccn%5d (accessed on 18 December 2025). (K-L0007), CP196517 Available online: https://www.ncbi.nlm.nih.gov/nuccore/CP196517 (accessed on 18 December 2025). (K-L0030), CP196519–CP196520 Available online: https://www.ncbi.nlm.nih.gov/nuccore/?term=CP196519:CP196520%5baccn%5d (accessed on 18 December 2025). (K-L0044), CP196529 Available online: https://www.ncbi.nlm.nih.gov/nuccore/CP196529 (accessed on 18 December 2025). (K-L0071), CP196531–CP196533 Available online: https://www.ncbi.nlm.nih.gov/nuccore/?term=CP196531:CP196533%5baccn%5d (accessed on 18 December 2025). (K-L0107), CP196538 Available online: https://www.ncbi.nlm.nih.gov/nuccore/CP196538 (accessed on 18 December 2025). (K-L0201), CP196541–CP196542 Available online: https://www.ncbi.nlm.nih.gov/nuccore/?term=CP196541:CP196542%5baccn%5d (accessed on 18 December 2025). (K-L0361), CP196545–CP196546 Available online: https://www.ncbi.nlm.nih.gov/nuccore/?term=CP196545:CP196546%5baccn%5d (accessed on 18 December 2025). (K-L0373), CP196547 Available online: https://www.ncbi.nlm.nih.gov/nuccore/CP196547 (accessed on 18 December 2025). (K-L0377), CP196544 Available online: https://www.ncbi.nlm.nih.gov/nuccore/CP196544 (accessed on 18 December 2025). (K-M0056), CP142089–CP142091 Available online: https://www.ncbi.nlm.nih.gov/nuccore/?term=CP142089:CP142091%5baccn%5d (accessed on 18 December 2025). (K-M0228), CP142097–CP142098 Available online: https://www.ncbi.nlm.nih.gov/nuccore/?term=CP142097:CP142098%5baccn%5d (accessed on 18 December 2025). (K-M0252), CP196543 Available online: https://www.ncbi.nlm.nih.gov/nuccore/CP196543 (accessed on 18 December 2025). (K-M0259), CP142092–CP142093 Available online: https://www.ncbi.nlm.nih.gov/nuccore/?term=CP142092:CP142093%5baccn%5d (accessed on 18 December 2025). (K-M0260), CP196539–CP196540 Available online: https://www.ncbi.nlm.nih.gov/nuccore/?term=CP196539:CP196540%5baccn%5d (accessed on 18 December 2025). (K-M0312), CP196534 Available online: https://www.ncbi.nlm.nih.gov/nuccore/CP196534 (accessed on 18 December 2025). (K-M0357), CP124750–CP124753 Available online: https://www.ncbi.nlm.nih.gov/nuccore/?term=CP124750:CP124753%5baccn%5d (accessed on 18 December 2025). (K-M0706).

## Supplementary Materials

The following supporting information can be downloaded at https://www.mdpi.com/article/10.3390/pathogens15020140/s1, Figure SA: TYGS calculated phylogenetic tree of the samples and the corresponding type strains. Figure SB: GTDB wf_de_novo calculated phylogenetic tree of the samples and the corresponding type strains visualized using iTOL. Figure SC: GTDB wf_de_novo calculated phylogenetic tree of the external samples and the corresponding type strains visualized using iTOL. Table SA: Pairwise calculation of dDDH values of the samples and the corresponding type strains. Table SB: GTDB analysis of the samples and the corresponding type strains. The calculations are performed using GTDB-Tk wf_classify. Table SC: List of plasmids for the samples, including the reference. Table SD: GTDB classification of external genomes for new classification. The calculations are performed using GTDB-Tk wf_classify. Table SE: Sample information list for genomes carrying the 83.7 kbp plasmid of interest.
